# Novel Chimeric Peptides Based on the Enolase Peptide Antigen (CEP-1) Bearing Three Post-Translational Modifications (Citrullination, Homocitrullination and Acetylation) for Determining the Diagnosis and Severity of Rheumatoid Arthritis

**DOI:** 10.3390/ijms251910654

**Published:** 2024-10-03

**Authors:** María José Gómara, Juan C. Sarmiento-Monroy, Raul Castellanos-Moreira, José A Gómez-Puerta, Raimon Sanmartí, Isabel Haro

**Affiliations:** 1 Unit of Synthesis and Biomedical Applications of Peptides, Institut de Química Avançada de Catalunya, Consejo Superior de Investigaciones Científicas (IQAC-CSIC), Jordi Girona 18-26, 08034 Barcelona, Spain; 2 Department of Rheumatology, Hospital Clínic of Barcelona, 08036 Barcelona, Spain; sarmiento@clinic.cat (J.C.S.-M.); castellanos@clinic.cat (R.C.-M.); jagomez@clinic.cat (J.A.G.-P.); sanmarti@clinic.cat (R.S.)

**Keywords:** rheumatoid arthritis, interstitial lung disease, diagnosis, chimeric peptides, synthetic peptides, fibrin, vimentin, filaggrin, enolase, citrullination, homocitrullination, acetylation

## Abstract

With the aim of improving the uncertainties associated with the correct diagnosis of seronegative rheumatoid arthritis (RA) and identifying those at risk of developing interstitial lung disease (ILD), we have designed new peptide antigens bearing three post-translational modifications (PTMs) (citrulline, homocitrulline and acetyl-lysine) related to RA that could complement existing tests based on anti-citrullinated peptide/protein antibodies (ACPAs). Several chimeric peptides were synthesized and comparatively tested as antigens in ELISAs with two cohorts of sera: 178 RAs and 110 healthy blood donors. The results indicated that although chimeric peptides containing all three PTMs and vimentin and enolase domains do not significantly outperform existing ACPA tests in terms of sensitivity and specificity, they show potential to complement current assays, especially when detecting antibodies in some seronegative patients. Furthermore, the presence of these autoantibodies significantly identified patients with RA and ILD. We can conclude that the identification of specific autoantibody profiles using synthetic antigens containing peptide domains derived from proteins present in the human joint could help in the early detection of the risk of ILD in patients with RA and be useful for adapting follow-up strategies and guiding decisions during treatment.

## 1. Introduction

Inflammatory rheumatic diseases are autoimmune and/or immune-mediated diseases frequently caused by the immune system’s own attack on the joints, muscles, bones and organs and account for a large percentage of disability [1]. Among them, rheumatoid arthritis (RA) is the most common, affecting 0.5–1% of adults worldwide with women being three times more susceptible than men. RA is a complex and heterogeneous disease which unless having prominent physical manifestation of arthritis and bone erosion is difficult to diagnose and treat, especially in the early development stages or in patients with seronegative status for autoantibodies according to the American College of Rheumatology (ACR)/European Alliance of Associations for Rheumatology (EULAR) classification criteria in 2010 [2]. For this reason, currently, it is still necessary to discover and develop new biomarkers to stratify patients into more homogeneous groups, with the aim of improving both diagnosis and therapeutic decisions [3,4].

The presence of anti-citrullinated peptide/proteins antibodies (ACPAs) in RA patients has been widely described as the most specific serological biomarker for RA. The current gold standard for their detection is commercial assays (CCP2 and CCP3) which are composed of peptides that are not present in the proteins at human joints [5,6]. With the aim of designing novel peptide antigens based on biologically relevant proteins in the RA context, in initial works, we synthesized and explored the diagnostic value of citrullinated chimeric peptide antigens derived from different proteins that are present in rheumatoid synovial fluid [7,8,9,10]. Our findings highlighted the use of these chimeric citrullinated peptides for RA diagnosis and indicated that more than one serological test is required to classify RA patients based on the presence or absence of ACPAs.

However, citrullination is not the only post-translational modification (PTM) related to RA; homocitrullination (carbamylation) and acetylation are also responsible for the generation of anti-modified protein/peptide antibodies (AMPA family), which are autoantibodies that have been implicated in the etiopathogenesis, diagnosis and prognosis of RA [4,11,12]. With this in mind, we have recently described for the first time a peptide-based antigen (namely chimeric fibrin filaggrin citrullinated homocitrullinated acetylated peptide, CFFCHAP) which simultaneously contains the three PTMs (citrullination, homocitrullination and acetylation) considered to be the most relevant for RA. Our results showed that patients with anti-CFFCHAP antibodies have a more severe RA phenotype in terms of joint destruction as well as the presence of interstitial lung disease (ILD), a severe extra-articular manifestation in RA patients that entails a high mortality [13].

ILD is indeed a serious complication of RA that affects 4–50% of RA patients, depending on the screening method and population studied. ILD is the leading cause of death in RA patients, accounting for up to 35% of RA-related deaths [14]. The significant mortality risk associated with RA-ILD underscores the importance of early diagnosis and appropriate management to improve patient outcomes.

Here, in the present work, we try to identify additional biomarkers considering other proteins such as α-enolase or vimentin that are also present in the rheumatoid synovium. With this aim of addressing unmet needs in RA diagnosis, we have designed novel chimeric peptides which have a common peptide sequence corresponding to the well-known α-enolase antigen CEP-1. Antibodies against CEP-1 are found in approximately 46% of serum samples from RA patients, and native alpha-enolase is abundantly expressed in the synovium of RA patients [15,16]. Recently, Haolong Li et al. conducted a systematic review and meta-analysis to evaluate the diagnostic performance of CEP-1. Although the authors concluded that anti-CEP-1 antibodies have a moderate RA diagnostic value compared to anti-CCP antibodies, which are routinely used to diagnose RA together with the rheumatoid factor (RF), some studies indicate that anti-CEP-1 could help to identify RA patients with anti-CCP-negative tests [17].

## 2. Results

**Figure 1:**
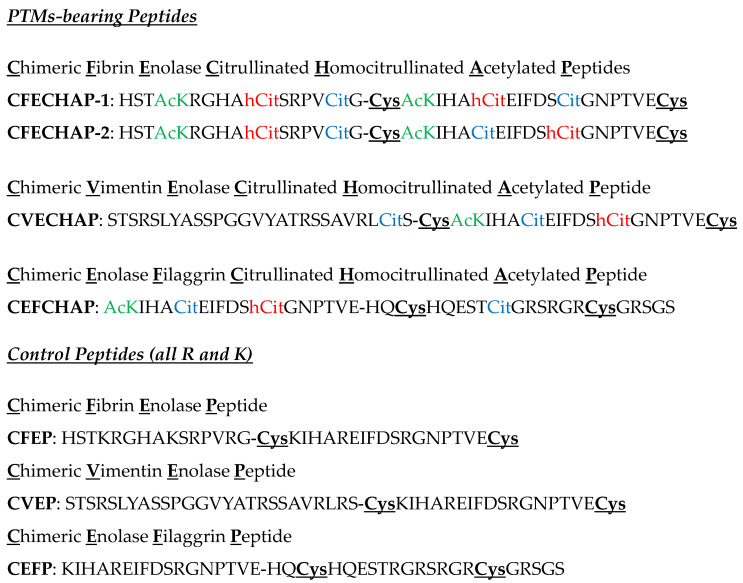
Primary structures of chimeric peptides. Cys, which forms the intramolecular disulfide bonds, is highlighted in bold and underlined. Citrulline in blue, Homocitrulline in red and Acetyl-Lysine in green.

With the aim of improving the uncertainties associated with the correct diagnosis of seronegative RA patients and those at risk of developing ILD, we designed novel peptide antigens (Figure 1) bearing three post-translational modifications (citrulline, homocitrulline and acetyl-lysine) related to this disease that could complement existing ACPA-based tests.

**Figure 2:**
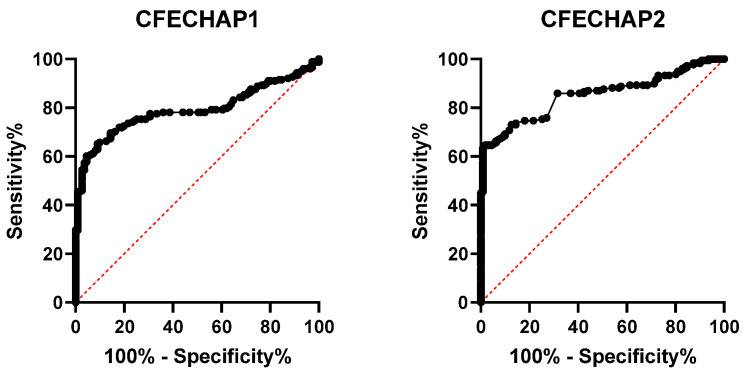
ROC curve analysis. ELISA results with chimeric fibrin/enolase peptides in the cohort of patients with RA (*n* = 178) and healthy blood donors (*n* = 110). Areas under the curve (AUCs) at 95% CI for CFECHAP-1 and CFECHAP-2 were 0.792 and 0.852, respectively.

Firstly, two cyclic chimeric fibrin/enolase peptides (CFECHAP-1 and CFECHAP-2) that differed on the position of citrulline and homocitrulline in the enolase sequence (CEP-1) were synthesized. Using CFECHAP-1 and CFECHAP-2 as coating antigens, a comparative ELISA was performed in cohorts of 178 RA and 110 BD sera. The values of the area under the ROC curve (AUC, RA vs. BD) are shown in Figure 2. In order to guarantee that the sera reactivity is specific for the post-translational modifications present on the peptide backbones, a cut-off ≥ 0.1 in optical density (OD) was established. The OD values result from subtracting the values of the unmodified peptides from the values of the PTM peptides. Taking into account the best results obtained for the CFECHAP-2 version of the two chimeric peptides derived from fibrin and enolase proteins in terms of the balance of sensitivity and specificity, as well as the AUC, next, we decided to synthesize two new chimerics based on vimentin/enolase (CVECHAP) and enolase/filaggrin (CEFCHAP) proteins, keeping the same PTM combinations that were present in the CFECHAP-2 peptide in the CEP-1 peptide (Figure 1).

Unmodified basal peptides in the Arg and Lys residues were also synthesized and cyclisized for their use as control peptides (CEFP, CVEP and CEFP) (Figure 1). Differently to CFECHAP-1, CFECHAP-2, CVECHAP, CFEP and CVEP peptides that have the disulfide bond at the CEP-1 antigen, in CEFCHAP and CEFP peptides, the intramolecular cyclization was performed between the Cys at the filaggrin sequence (highlighted in bold and underlined). All the peptides were purified by reverse-phase, high-performance liquid chromatography (RP-HPLC) to ≥ 95% purity. Table 1 shows the capacity factor (k’) calculated by RP-HPLC and the mass calculated and found by electrospray ionization mass spectrometry (ESI-MS).

**Figure 3:**
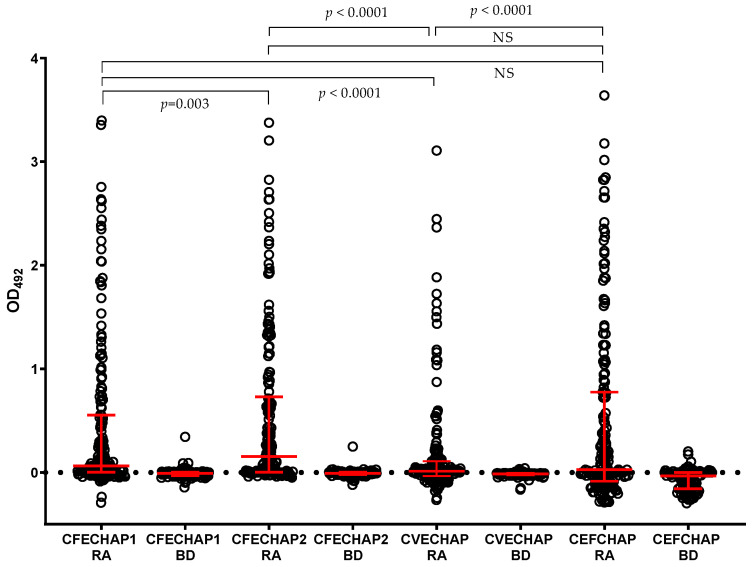
AMPA antibody levels in RA patients (*n* = 178) and healthy blood donors (BDs) (*n* = 110) for each chimeric enolase peptide. Red lines represent the median and interquartile range. All antibody levels were significantly higher (*p* < 0.0001) in RA patients compared to BDs for each chimeric peptide using Mann–Whitney U test. The statistical significance between RA groups was analyzed by the parametric paired *t* test and the values are shown in the figure. NS, non-significant.

The different chimeric enolase peptides were comparatively tested as antigens in ELISAs with the two cohorts of 178 RA and 110 BD sera. The AMPA reactivity is shown in Figure 3. The results demonstrated that reactivity in RA was significantly higher than in healthy blood donors for each peptide (*p* < 0.0001). Thus, healthy blood donors displayed none or very limited reactivity in the assays. When comparing the AMPA reactivity anti-chimeric enolase peptides in RA patients, we observed significant differences between antibody levels in anti-fibrin/enolase or enolase/filaggrin and anti-vimentin/enolase peptides, while the differences in antibody levels were not significant between fibrin/enolase and enolase/filaggrin peptides (Figure 3).

The results of the ROC curves as well as the sensitivity and specificity for each chimeric enolase-based peptide are shown in Table 2 and Figure S1.

**Figure 4:**
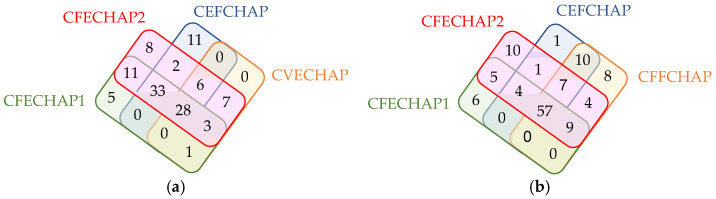
Venn diagram for positivity to the chimeric enolase peptides in 178 RA patients (**a**) and Venn diagram for positivity to the chimeric peptides based on filaggrin and/or fibrin sequences in 178 RA patients (**b**).

Figure 4a shows the overlapping reactivity between the four chimeric enolase peptides in 178 RA patients. While 28 RA sera tested positive for the four chimeric enolase peptides, a greater number of sera (33) were positive for the chimeric peptides that are composed of the filaggrin or fibrin sequences covalently linked to the CEP-1 peptide (CFECHAP-1, CFECHAP-2 and CEFCHAP). The chimeric CVECHAP peptide based on vimentin/enolase sequences showed the lowest reactivity, while the CFECHAP-2 peptide showed the highest overlapping positivity. Figure 4b shows the overlapping reactivity between the chimeric peptides containing the fibrin and/or filaggrin sequences in their structure. A total of 57 RA sera were positive for the chimeric peptides based on fibrin/enolase, enolase/filaggrin and fibrin/filaggrin, demonstrating the similarities between them. The fibrin/enolase-based peptides CFECHAP-1 and/or CFECHAP-2 overlapped in 13 positive sera with the CFFCHAP, while the enolase/filaggrin-based CEFCHAP overlapped in 10 positive sera with the chimeric fibrin/filaggrin peptide. Therefore, it appears that the similarity in reactivity of chimeric peptides based on the fibrin sequence is greater than that of chimeric filaggrin peptides. However, it should be noted that there were a total of 27 RA sera negatively tested by CFFCHAP that were reactive for the chimeric peptides based on fibrin/enolase and/or enolase/filaggrin (CFECHAP-1, CFECHAP-2 and CEFCHAP).

Based on previous studies that indicate that anti-CEP-1 could help to identify ACPA-negative RA patients, we analyzed the reactivity of chimeric enolase peptides with the ACPA-seronegative RA sera (*n* = 51) from the 178 RA patients. Table 3 shows the number of established RA sera detected by the different chimeric enolase peptides classified as ACPA-positive or -negative. We also included in this table the previously reported chimeric fibrin/filaggrin peptide (CFFCHAP). Analyzing the set of chimeric peptides, nine additional ACPA-negative sera (17.6%) were identified. Out of these nine sera, five sera (9.8%) were not identified by the fibrin/filaggrin chimeric peptide, indicating the additional diagnostic value in ACPA-negative RA by combining analysis of chimeric enolase peptides.

Taking into account that RA-ILD has been associated not only with different anti-modified protein antibodies based on citrulline [18,19,20], homocitrulline [21] and malondialdehyde–acetaldehyde adducts [22] but also with a peptide antigen bearing multiple PTMs that we previously designed [13], the novel chimeric sequences reported here were also analyzed regarding the status of RA patients: RA-ILD vs. RA-non-ILD, degree of joint destruction and the presence of an erosive disease.

As shown in Table 4, autoantibodies were more frequent in the RA-ILD group of patients (anti-CFECHAP-1 51.3% vs. 44.0%; anti-CFECHAP-2 75.7% vs. 48.9%; anti-CVECHAP 27.0% vs. 24.8%; anti-CEFCHAP 56.8% vs. 41.8%; anti-CFFCHAP 64.9% vs. 50.4%). Of note, this difference was only statistically significant for the chimeric containing fibrin and enolase domains CFECHAP-2 (*p* = 0.0036). Regarding the titers, although a trend to associate the presence of anti-CFECHAP-2 median titers with ILD (*p* = 0.064), the differences were only significant for CFECHAP-1 and the chimeric containing fibrin and filaggrin peptides (CFFCHAPs) that we previously reported [13].

The severity of joint damage was scored according to the modified Larsen method [23]. As shown in Table 5, regarding the novel chimerics containing the CEP-1 domain, only a clear association was observed for CEFCHAP, with a higher prevalence of autoantibodies in patients with a more severe disease (Larsen ≥ 18) in comparison to those with mild joint destruction (Larsen < 18). The analyses of the comparison of autoantibodies presenting erosive RA did not render statistically significant results in any case (Table S1).

## 3. Discussion

Improving the uncertainties associated with the diagnosis of seronegative RA patients as well as identifying those at risk of developing ILD are key challenges and unmet needs in clinical practice. For a higher proportion of correct diagnoses and risk stratification, other nontraditional autoantibodies need to be studied. Factors such as smoking, RF and ACPA seropositivity have been associated with an increasing risk of developing RA, but more studies are needed to develop putative predictive models [24]. Recently, we described for the first time in the literature a peptide-based antigen bearing citrulline, homocitrulline and acetyl-lysine within its sequence that was formed by domains of fibrin and filaggrin proteins and associates with the clinical phenotype of RA patients, specifically erosive disease with radiological structural damage and ILD [13]. In the present work, several novel antigen peptides bearing these three PTMs relevant for RA but considering other proteins that are present in the rheumatoid synovial tissue, such as alpha-enolase or vimentin, have been designed and synthesized trying to address the still unmet need of identifying those RA patients who will develop ILD.

The results of this study indicate that while the new chimeric peptides bearing the three PTMs and containing domains from vimentin and enolase (CFECHAP, CVECHAP and CFECHAP) did not significantly improve the overall sensitivity/specificity balance compared to the fibrin/filaggrin chimeric peptide (CFFCHAP), they did show some potential benefits. Firstly, they have a complementary diagnostic value, since some anti-ACPA-negative sera tested positive with the new chimeric peptides, suggesting they may help identify additional RA cases that would be missed by existing tests. Secondly, by incorporating domains from vimentin and enolase, the resulting chimeric peptides expand the range of epitopes that can be detected, which may capture more diverse autoantibody responses in RA patients. Thus, the varied responses to different chimeric peptides suggest they could be useful for creating more detailed autoantibody profiles and capture more diverse autoantibody responses in individual RA patients. In summary, while the chimeric peptides do not significantly outperform existing ACPA tests in terms of sensitivity and specificity, they show potential for complementing current assays, especially for detecting antibodies in some seronegative samples. Another main advantage appears to be the ability to profile multiple epitopes simultaneously in a multiplex format, as previously reported for some chimeric citrullinated peptides [10].

Several studies have also indicated significant associations between high levels of anti-CEP-1 with erosions and ILD, supporting the notion that these autoantibodies may play a pathogenic role in RA’s severe extra-articular clinical manifestations [20,25]. The study by Alunno et al. found that anti-CEP-1 single positivity and anti-CCP/anti-CEP-1 double positivity, but not anti-CCP single positivity, were associated with ILD in RA patients [20]. In our study, the presence of autoantibodies against chimeric peptides containing both enolase and fibrin domains, specifically the CFECHAP-2 peptide, was found to significantly identify RA patients with ILD. This suggests that these chimeric peptides may serve as useful biomarkers for detecting ILD in RA patients. In addition, autoantibodies against chimerics containing the fibrin domain in their structure (CFECHAP-1 and CFFCHAP) showed significant differences in titers between RA patients with and without ILD. This highlights the importance of including the fibrin-derived peptide in chimeric constructs for distinguishing RA-ILD. However, chimeric peptides lacking the fibrin-derived component (CVECHAP and CEFCHAP) did not show significant differences between RA-ILD and RA-non-ILD groups. Finally, while the association with erosive changes or joint destruction was higher for different chimeric peptides, it did not exceed the significance achieved by the CFFCHAP chimeric. This suggests that these antibodies may have some relation to joint damage, but their primary utility may be in identifying ILD risk.

These findings underscore the complexity of autoantibody responses in RA and highlight the potential of using multiple antibody specificities to predict disease features and outcomes. Further research, particularly longitudinal studies, is needed to fully elucidate the predictive value of these antibodies for ILD development in RA patients.

## 4. Materials and Methods

### 4.1. Synthesis of Chimeric Peptides

The chimeric peptides shown in Figure 1 were synthesized manually in polypropylene syringes (Bond Elut, Agilent, Santa Clara, CA, USA), each fitted with a polyethylene porous disk. A PEG-PS copolymer functionalized with the modified Rink linker to a extent of 0.19 mmol/g (Novasyn^®^ TGR resin, Novabiochem, Merck Millipore, Oakville, ON, Canada) was used for the synthesis of carboxamides peptides. Activation of the carboxylic group of 9-fluorenylmethoxycarbonyl (Fmoc)-protected amino acids (Novabiochem, Merck Millipore, Oakville, ON, Canada) (3 equiv.) was carried out by addition of 2-(1H-7-azabenzotriazole-1-yl)-1,1,3,3-tetramethyluronium hexafluorophosphate methanaminium (HATU, Genscript, Piscataway, NJ, USA) (3 equiv.) and diisopropylethylamine (DIEA, Sigma-Aldrich, Merck KGaA, Darmstadt, Germany) (6 equiv.) in dimethylformamide (DMF, Scharlau, Sentmenat, Spain). The mixture was added to the resin and allowed to react with intermittent manual stirring for 30 min. The solvent was removed by filtration, and the resin was washed with DMF (5 × 30 s). The extent of coupling was checked by the Kaiser colorimetric assay. The Fmoc group was removed by treating the resin with 20% piperidine in DMF (3–4 mL/g resin, 2 × 10 min). The peptide elongation continued by coupling the second amino acid and the following amino acids with the same procedure. Once the peptide sequences were completed, the peptidyl resins were biotinylated at the N-terminus by addition of N-biotinyl-NH-(PEG)_2_-COOH (4 equivalents) activated with benzotriazole-1-oxytris(pirrolidino) phosphonium hexafluorophosphate (PyBOP) (4 equivalents), 1-hydroxybenzotriazole (HOBt) (4 equivalents) and DIEA (8 equivalents. The reactions were left overnight and were checked by the ninhydrin colorimetric reaction. All the reagents used for the biotinylation were from Sigma-Aldrich, Merck KGaA, Darmstadt, Germany.

The peptides were cleaved from the resin by means of treatment with 95% trifluroacetic acid (TFA) in the presence of scavengers, basically 2% (*v*/*v*) H_2_O, 2% (*v*/*v*) 2-mercaptoethanol and 1% (*v*/*v*) triisopropylsilane (TIS) for 4 h. In order to synthesize the cyclic chimeric peptide, 2 serine residues in the linear peptides were substituted by 2 residues of Fmoc-S-acetamidomethyl-L-cysteine for their subsequent oxidation and formation of disulfide bonds. After peptide cleavage, the peptide was dissolved in acetic acid/H_2_O (1:1, 3 mg/mL) under N_2_, then HCl (1 M, 0.1 mL/mg) followed by I_2_ (20 equiv./Acm) were added. After 4 h, I_2_ was quenched by adding 1M ascorbic acid drop-wise until the mixture became colorless and was concentrated by evaporation under reduced pressure to approximately one-third of the original volume.

The final products were purified by semipreparative RP-HPLC on an Agilent Technologies 1260 Infinity chromatograph using an Agilent ZORBAX^®^ SB-C18 (semi-preparative RP, 9.4 × 250 mm, particle size 5 µm) (Agilent Technologies, Santa Clara, CA, USA). A linear gradient of 100–50% A in B was achieved over 30 min at a flow rate of 2.5 mL/min using 0.05% TFA in water (A) and 0.05% TFA in acetonitrile (B) as an eluting system. The purified biotinylated chimeric peptides (purity higher that 95%) were characterized by analytical RP-HPLC and ESI-MS (Table 1 and Figure S1 Supplementary Material) on a Waters ACQUITY Arc Multi-Dimensional Liquid Chromatograph (MDLC) (Waters Corporation, Mildford, CT, USA) coupled to a Photo Diode Array (Waters PDA 2998) detector, an Evaporative Light-Scattering (Waters ELS 2424) detector and a Single Quadrupol mass detector (Waters SQ Detector 2). Analytical RP-HPLC was performed on a ZORBAX^®^ RR Extent C18 column (2.1 × 50 mm, particle size 3.5 µm) with a gradient of 95–5% A in B over 5 min at a flow rate of 0.7 mL/min using 0.05% formic acid in water (A) and 0.05% formic acid in acetonitrile (B) as an eluting system. Characterizations of the final peptides are shown in Figures S1–S7.

### 4.2. Serum Samples

A cross-sectional study including RA patients diagnosed according to the 2010 ACR/EULAR criteria assessed in a rheumatology department outpatient clinic of a tertiary university hospital was performed. Individuals fulfilling other inflammatory arthritis or connective tissue disease diagnostic criteria were excluded. The study population includes consecutive patients diagnosed with RA-ILD and RA without ILD. RA-ILD was diagnosed using high-resolution computed tomography and confirmed by a multidisciplinary committee. Clinical, demographic and therapeutic characteristics were evaluated (Table 6). More detailed information about the study population and inclusion criteria are described elsewhere [21]. To obtain ROC curves, 110 healthy blood donors of the same hospital without inflammatory rheumatic diseases were also analyzed.

### 4.3. ELISAs

Nunc MaxiSorp microtiter plates were incubated with Neutravidin protein (Thermo Fisher Scientific) diluted in phosphate-buffered saline (PBS) (0.5 μg/well) overnight at 4 °C and, thereafter, for 1 h at 37 °C. After washing the plates, the biotinyl peptides were diluted at 1 μg/mL in PBS and 100 μL of the peptide solution was added to each well. The plates were incubated for 1 h at 37 °C. Subsequently, the plates were blocked with 2% bovine serum albumin (BSA) in PBS with 0.05% Tween-20 for 30 min at 37 °C. Then, the plates were washed 3 times. Sera were diluted 250-fold in RIA buffer (1% BSA wt/v, 350 mM NaCl, 10 mM TRIS, 1% *v*/*v* Triton X-100, 0.5% wt/v Na-deoxycholate and 0.1% wt/v SDS) supplemented with 10% fetal bovine serum and 100 μL of the dilution was added to each well. The plates were incubated for 1 h at 37 °C and then overnight at 4 °C. Afterwards, each plate was washed 3 times with PBS/0.05% Tween-20 and 100 μL of anti-human IgG secondary antibody conjugated to peroxidase (peroxidase-conjugated rabbit anti-human IgG specific for gamma-chains, Dako, Glostrup, Denmark) diluted in RIA buffer at 1:4000 was added to each well and incubated for 1 h at 37 °C. After washing the plates, detection of bound antibodies was carried out using SigmaFast (Sigma-Aldrich, St. Louis, MO, USA), with o-phenylenediamine dihydrochloride (OPD) as a substrate. The reaction was stopped with 50 μL of 2N H_2_SO_4_ and plates were read at 492 nm. All sera were tested in duplicate. Reactivity to the unmodified basal peptides’ structures (CFEP, CVEP and CEFP) was subtracted from the reactivity to CFECHAP-1, CFECHAP-2, CVECHAP and CEFCHAP, respectively (citrullinated, homocitrullinated and acetylated peptides) to ensure that the measured reactivity shown was specific to the post-translational modifications under study. Results were obtained in values of optical density (OD) and ranged from 0 to 4, where 0 implies no reactivity and 4 is the highest value of intensity of reactivity.

### 4.4. Statistical Analysis

The performance of the chimeric peptides in the diagnosis of RA samples was analyzed using receiver-operating characteristic (ROC) curves. Continuous variables were presented as median and interquartile range. Differences between continuous variables of RA and blood donors groups were analyzed by using the non-parametric Mann–Whitney U test. Differences between continuous variables of RA groups were analyzed by the parametric paired *t* test. Statistical significance was established as two-tailed *p*-values < 0.05 in all analyses. All the statistical analyses were performed using GraphPad Prism 8.

### 4.5. Ethics

Signed informed consent was obtained from all patients before study enrolment. The Strengthening the Reporting of Observational Studies in Epidemiology guidelines were followed.

## Figures and Tables

**Table 1:** RP-HPLC and ESI-MS characterization of biotinyl-PEG_2_-chimeric peptides.

Biotinyl-PEG_2_-Peptide	^1^ RP-HPLC (k’)	^2^ Mass Calcd.	^2^ Mass Found
CFEP	4.4	4369.2	4368.6
CFECHAP-1	5.1	4513.2	4513.9
CFECHAP-2	4.9	4513.2	4514.2
CVEP	5.0	5411.7	4512.1
CVECHAP	6.7	5474.1	5474.2
CEFP	5.5	4833.3	4832.8
CEFCHAP	5.6	4892.3	4891.5

^1^ Capacity factor (k’) was calculated by RP-HPLC. ^2^ Masses calculated and found by low-resolution mass spectrometry (LRMS) were obtained by ESI-MS.

**Table 2:** Sensitivity and specificity at the cut-off at optical density ≥0.1 and AUC (95% CI) of the ROC curves.

Peptides	Sensitivity	95% CI	Specificity	95% CI	LR	AUC
CFECHAP-1	44.94	37.82–52.28	99.10	95.07–99.95	49.89	0.792 (0.740–0.844)
CFECHAP-2	53.93	46.60–61.09	99.10	95.07–99.95	59.87	0.852 (0.809–0.896)
CVECHAP	25.28	19.46–32.14	100.0	96.65–100.0	–	0.687 (0.623–0.751)
CEFCHAP	44.94	37.82–52.28	97.30	92.35–99.26	16.63	0.763 (0.710–0.816)

AUC, area under the curve; CI, confidence interval; LR, likelihood ratio.

**Table 3:** AMPA reactivity and frequencies of chimeric peptides in RA patients.

AMPA IgG Specificity	RA Sera (*n* = 178)	ACPA-Positive (*n* = 127)	ACPA-Negative (*n* = 51)
CFECHAP-1	81 (45.5%)	78 (61.4%)	3 (5.9%)
CFECHAP-2	97 (54.5%)	94 (74%)	3 (5.9%)
CVECHAP	45 (25.3%)	44 (34.6%)	1 (2.0%)
CEFCHAP	80 (44.9%)	79 (62.2%)	1 (2.0%)
CFFCHAP	95 (53.4%)	91 (71.7%)	4 (7.8%)
Any peptide	122 (68.5%)	113 (89.0%)	9 (17.6%)

**Table 4:** Autoantibody status in RA patients (ILD vs. non-ILD).

	RA-ILD (*n* = 37)	RA-non-ILD (*n* = 141)	*p*-Value
anti-CFECHAP-1-positive (%)	19 (51.3)	62 (44.0)	NS
median titer anti-CFECHAP-1 (IQR)	0.131 (0.861)	0.055 (0.522)	0.015
anti-CFECHAP-2-positive (%)	28 (75.7)	69 (48.9)	0.0036
median titer anti-CFECHAP-2 (IQR)	0.352 (0.987)	0.075 (0.649)	NS
anti-CVECHAP-positive (%)	10 (27.0)	35 (24.8)	NS
median titer anti-CVECHAP (IQR)	0.017 (0.133)	0.009 (0.104)	NS
anti-CEFCHAP-positive (%)	21 (56.8)	59 (41.8)	NS
median titer anti-CEFCHAP (IQR)	0.268 (1.880)	0.001 (0.534)	NS
anti-CFFCHAP-positive (%)	24 (64.9)	71 (50.4)	NS
median titer anti-CFFCHAP (IQR)	0.425 (2.811)	0.108 (1.013)	0.026
anti-CCP3-positive (%)	29 (78.4)	98 (69.5)	NS
median titer anti-CCP3 AU/mL (IQR)	674 (2215)	143 (1139)	NS

NS, non-significant.

**Table 5:** Comparison of the presence of autoantibodies with the degree of joint destruction according to the modified Larsen score.

Median Larsen Score 18	Larsen < 18 (*n* = 88)	Larsen ≥ 18 (*n* = 90)	*p*-Value
anti-CFECHAP-1-positive (%)	37 (42.0)	44 (48.9)	NS
median titer anti-CFECHAP-1 (IQR)	0.048 (0.326)	0.080 (0.687)	NS
anti-CFECHAP-2-positive (%)	42 (47.7)	55 (61.1)	NS
median titer anti-CFECHAP-2 (IQR)	0.071 (0.631)	0.274 (0.767)	NS
anti-CVECHAP-positive (%)	21 (23.9)	24 (26.7)	NS
median titer anti-CVECHAP (IQR)	0.009 (0.084)	0.012 (0.109)	NS
anti-CEFCHAP-positive (%)	33 (37.5)	47 (52.2)	0.048
median titer anti-CEFCHAP (IQR)	0.000 (0.424)	0.162 (1.105)	0.0274
anti-CFFCHAP-positive (%)	40 (45.4)	55 (61.1)	0.036
median titer anti-CFFCHAP (IQR)	0.058 (0.527)	0.285 (2.014)	0.0073
anti-CCP3-positive (%)	60 (68.2)	67 (74.4)	NS
median titer anti-CCP3 AU/mL (IQR)	104 (1011)	454 (2015)	0.025

NS, non-significant.

**Table 6:** Demographic, clinical and therapeutic characteristics of RA patients.

Female (%)	141 (79)
Age mean (±SD)	59.7 (13.0)
Mean disease duration (±SD)	6.6 (5.0)
Ever smokers (%)	83 (46)
Caucasian (%)	151 (84)
Rheumatoid factor-positive	111 (62)
Extra-articular manifestations	
Sicca syndrome	33 (18)
Rheumatoid nodules	21 (12)
Serositis	3 (2)
Treatment	
Glucocorticoids (%)	108 (60)
csDMARDs (%)	155 (87)
MTX (%)	115 (64)
bDMARDs (%)	47 (26)
Mean DAS 28 (±SD)	2.94 (1.18)
Radiographic erosive disease (%)	89 (50)

csDMARDS: conventional synthetic disease-modifying antirheumatic drugs, MTX: methotrexate, bDMARDS: biological DMARDS, DAS: disease activity score.

## Data Availability

Data are contained within the article or Supplementary Materials. The data presented in this study are available on request from the corresponding author.

## Supplementary Materials

The following supporting information can be downloaded at https://www.mdpi.com/article/10.3390/ijms251910654/s1.

## References

[1] Sangha O. (2000). Epidemiology of rheumatic diseases. Rheumatology.

[2] Aletaha D., Neogi T., Silman A.J., Funovits J., Felson D.T., Bingham C.O., Birnbaum N.S., Burmester G.R., Bykerk V.P., Cohen M.D. (2010). Rheumatoid arthritis classification criteria: An American College of Rheumatology/European League Against Rheumatism collaborative initiative. Ann. Rheum. Dis..

[3] Conti V., Corbi G., Costantino M., De Bellis E., Manzo V., Sellitto C., Stefanelli B., Colucci F., Filippelli A. (2020). Biomarkers to Personalize the Treatment of Rheumatoid Arthritis: Focus on Autoantibodies and Pharmacogenetics. Biomolecules.

[4] Haro I., Sanmartí R., Gómara M.J. (2022). Implications of Post-Translational Modifications in Autoimmunity with Emphasis on Citrullination, Homocitrullination and Acetylation for the Pathogenesis, Diagnosis and Prognosis of Rheumatoid Arthritis. Int. J. Mol. Sci..

[5] van Venrooij W.J., Zendman A.J. (2008). Anti-CCP2 antibodies: An overview and perspective of the diagnostic abilities of this serological marker for early rheumatoid arthritis. Clin. Rev. Allergy Immunol..

[6] Vos I., Van Mol C., Trouw L.A., Mahler M., Bakker J.A., Van Offel J., De Clerck L., Huizinga T.W. (2017). Anti-citrullinated protein antibodies in the diagnosis of rheumatoid arthritis (RA): Diagnostic performance of automated anti-CCP-2 and anti-CCP-3 antibodies assays. Clin. Rheumatol..

[7] Pérez M.L., Gómara M.J., Ercilla G., Sanmartí R., Haro I. (2007). Antibodies to citrullinated human fibrinogen synthetic peptides in diagnosing rheumatoid arthritis. J. Med. Chem..

[8] Sanmartí R., Graell E., Pérez M.L., Ercilla G., Viñas O., Gómez-Puerta J.A., Gratacós J., Balsa A., Gómara M.J., Larrosa M. (2009). Antibodies against chimeric fibrin/filaggrin citrullinated synthetic peptides in rheumatoid arthritis. Diagnostic and prognostic value. Arthritis Res. Ther..

[9] Malakoutihak M., Gómara M.J., Gómez-Puerta J.A., Sanmartí R., Haro I. (2011). The use of chimeric vimentin citrullinated peptides for the diagnosis of rheumatoid arthritis. J. Med. Chem.

[10] García-Moreno C., Gómara M.J., Bleda M.J., Sanmartí R., Haro I. (2019). A multiplex assay based on chimeric citrullinated peptides for the diagnosis of rheumatoid arthritis. PLoS ONE.

[11] van der Woude D., Toes R.E.M. (2024). Immune response to post-translationally modified proteins in rheumatoid arthritis: What makes it special?. Ann. Rheum. Dis..

[12] Kwon E.-J., Ju J.H. (2021). Impact of Posttranslational Modification in Pathogenesis of Rheumatoid Arthritis: Focusing on Citrullination, Carbamylation, and Acetylation. Int. J. Mol. Sci..

[13] García-Moreno C., Gómara M.J., Castellanos-Moreira R., Sanmartí R., Haro I. (2021). Peptides bearing multiple post-translational modifications as antigenic targets for severe rheumatoid arthritis patients. Int. J. Mol. Sci..

[14] Hyldgaard C., Hilberg O., Pedersen A.B., Ulrichsen S.P., Løkke A., Bendstrup E., Ellingsen T. (2017). A population-based cohort study of rheuma-toid arthritis-associated interstitial lung disease: Comorbidity and mortality. Ann. Rheum. Dis..

[15] Kinloch A., Tatzer V., Wait R., Peston D., Lundberg K., Donatien P., Moyes D., Taylor P.C., Venables P.J. (2005). Identification of citrullinated alpha-enolase as a candidate autoantigen in rheumatoid arthritis. Arthritis Res. Ther..

[16] Lundberg K., Kinloch A., Fisher B.A., Wegner N., Wait R., Charles P., Mikuls T.R., Venables P.J. (2008). Antibodies to citrullinated alpha-enolase peptide 1 are specific for rheumatoid arthritis and cross-react with bacterial enolase. Arthritis Rheum..

[17] Li H., Li L., Liu C., Cheng L., Yan S., Chen H., Li Y. (2021). Diagnostic value of anti-citrullinated α-enolase peptide 1 antibody in patients with rheumatoid arthritis: A systematic review and meta-analysis. Int. J. Rheum. Dis..

[18] Solomon J.J., Matson S., Kelmenson L.B., Chung J.H., Hobbs S.B., Rosas I.O., Dellaripa P.F., Doyle T.J., Poli S., Esposito A.J. (2020). IgA antibodies directed against citrullinated protein antigens are elevated in patients with idiopathic pulmonary fibrosis. Chest.

[19] Harlow L., Rosas I.O., Gochuico B.R., Mikuls T.R., Dellaripa P.F., Oddis C.V., Ascherman D.P. (2013). Identification of citrullinated Hsp90 isoforms as novel autoantigens in rheumatoid arthritis-associated interstitial lung disease. Arthritis Rheum..

[20] Alunno A., Bistoni O., Pratesi F., La Paglia G.M.C., Puxeddu I., Migliorini P., Gerli R. (2018). Anti-citrullinated alpha enolase antibodies, interstitial lung disease and bone erosion in rheumatoid arthritis. Rheumatology.

[21] Castellanos-Moreira R., Rodriguez-Garcia S.C., Gomara M.J., Ruiz-Esquide V., Cuervo A., Casafont-Sole I., Ramirez J., Holgado S., Gomez-puerta J.A., Cañete J.D. (2020). Anti-carbamylated proteins antibody repertoire in rheumatoid arthritis: Evidence of a new autoantibody linked to interstitial lung disease. Ann. Rheum. Dis..

[22] England B.R., Duryee M.J., Roul P., Mahajan T.D., Singh N., Poole J.A., Ascherman D.P., Caplan L., Demoruelle M.K., Deane K.D. (2019). Malondialdehyde–Acetaldehyde adducts and antibody responses in rheumatoid arthritis–associated interstitial lung disease. Arthritis Rheumatol..

[23] Kirwan J.R. (2000). Using the Larsen index to assess radiographic progression in rheumatoid arthritis. J. Rheumatol..

[24] Stainer A., Tonutti A., De Santis M., Amati F., Ceribelli A., Bongiovanni G., Torrisi C., Iacopino A., Mangiameli G., Aliberti S. (2023). Unmet needs and perspectives in rheumatoid arthritis-associated interstitial lung disease: A critical review. Front. Med..

[25] Liu Y., Liu C., Li L., Zhang F., Li Y., Zhang S. (2019). High levels of antibodies to citrullinated α-enolase peptide-1 (CEP-1) identify erosions and interstitial lung disease (ILD) in a Chinese rheumatoid arthritis cohort. Clin. Immunol..

