# Supplementary material for: Novel Chimeric Peptides Based on the Enolase Peptide Antigen (CEP-1) Bearing Three Post-Translational Modifications (Citrullination, Homocitrullination and Acetylation) for Determining the Diagnosis and Severity of Rheumatoid Arthritis
Source: Int J Mol Sci. 2024 Oct 3;25(19):10654. doi: 10.3390/ijms251910654 (PMC11477004; doi:10.3390/ijms251910654)
Supplement: Supplementary file 1 [file ijms-25-10654-s001.zip › ijms-3233063-supplementary.pdf]

Novel chimeric peptides based on the enolase peptide antigen (CEP-1) bearing three post-translational modifications (citrullination, homocitrullination and acetylation) for the diagnosis and severity of Rheumatoid Arthritis

María José Gómara 1,*, Juan C. Sarmiento-Monroy 2, Raul Castellanos-Moreira 2, José A Gómez-Puerta 2, Raimon Sanmartí 2 and Isabel Haro 1,*

[1] Unit of Synthesis and Biomedical Applications of Peptides, Institut de Química Avançada de Catalunya. Consejo Superior de Investigaciones Científicas (IQAC-CSIC), Jordi Girona 18-26, 08034 Barcelona, Spain
[2] Department of Rheumatology, Hospital Clínic of Barcelona, 08036 Barcelona, Spain; sarmiento@clinic.cat (J.C.S.-M.); castellanos@clinic.cat (R.C.-M.); jagomez@clinic.cat (J.A.G.-P.); sanmarti@clinic.cat (R.S.)
* Correspondence mariajose.gomara@iqac.csic.es (M.J.G.); isabel.haro@iqac.csic.es (I.H.)

Supplementary Material:

Table S1. Comparison of autoantibodies presenting erosive-RA (yes or not)

Figure S1. Primary structure, chemical formula, molecular weight, RP-HPLC chromatogram and ESI-MS spectrum of the **CFECHAP-1**

Figure S2. Primary structure, chemical formula, molecular weight, RP-HPLC chromatogram and ESI-MS spectrum of the **CFECHAP-2**

Figure S3. Primary structure, chemical formula, molecular weight, RP-HPLC chromatogram and ESI-MS spectrum of the **CVECHAP**

Figure S4. Primary structure, chemical formula, molecular weight, RP-HPLC chromatogram and ESI-MS spectrum of the **CEFCHAP**

Figure S5. Primary structure, chemical formula, molecular weight, RP-HPLC chromatogram and ESI-MS spectrum of the **CFEP**

Figure S6. Primary structure, chemical formula, molecular weight, RP-HPLC chromatogram and ESI-MS spectrum of the **CVEP**

Figure S7. Primary structure, chemical formula, molecular weight, RP-HPLC chromatogram and ESI-MS spectrum of the **CEFP**

Table S1. Comparison of autoantibodies presenting erosive-RA (yes or not)

	Erosive (n=89)	non-Erosive (n=89)	p value
**anti-CFECHAP-1 positive (%)**	40 (44.9)	41 (46.1)	NS
**median titer anti-CFECHAP-1 AU/mL (IQR)**	0.055 (0.628)	0.062 (0.481)	NS
**anti-CFECHAP-2 positive (%)**	52 (58.4)	45 (50.5)	NS
**median titer anti-CFECHAP-2 AU/mL (IQR)**	0.188 (0.893)	0.102 (0.685)	NS
**anti-CVECHAP positive (%)**	22 (24.7)	23 (25.8)	NS
**median titer anti-CVECHAP AU/mL (IQR)**	0.010 (0.104)	0.015 (0.112)	NS
**anti-CEFCHAP positive (%)**	40 (44.9)	40 (44.9)	NS
**median titer anti-CEFCHAP AU/mL (IQR)**	0.018 (0.855)	0.028 (0.724)	NS
**anti-CFFCHAP positive (%)**	47 (52.8)	48 (53.9)	NS
**median titer anti-CFFCHAP AU/mL (IQR)**	0.138 (1.617)	0.028 (0.724)	NS

Biotinyl-PEG$_2$-HSTAcKRGHAhCitSRPVCitG-**Cys**AcKIHAhCitEIFDSCitGNPTVE**Cys** (Cyclic)

Chemical formula: $C_{191}H_{310}N_{62}O_{59}S_3$
MW: 4515.08 g/mol

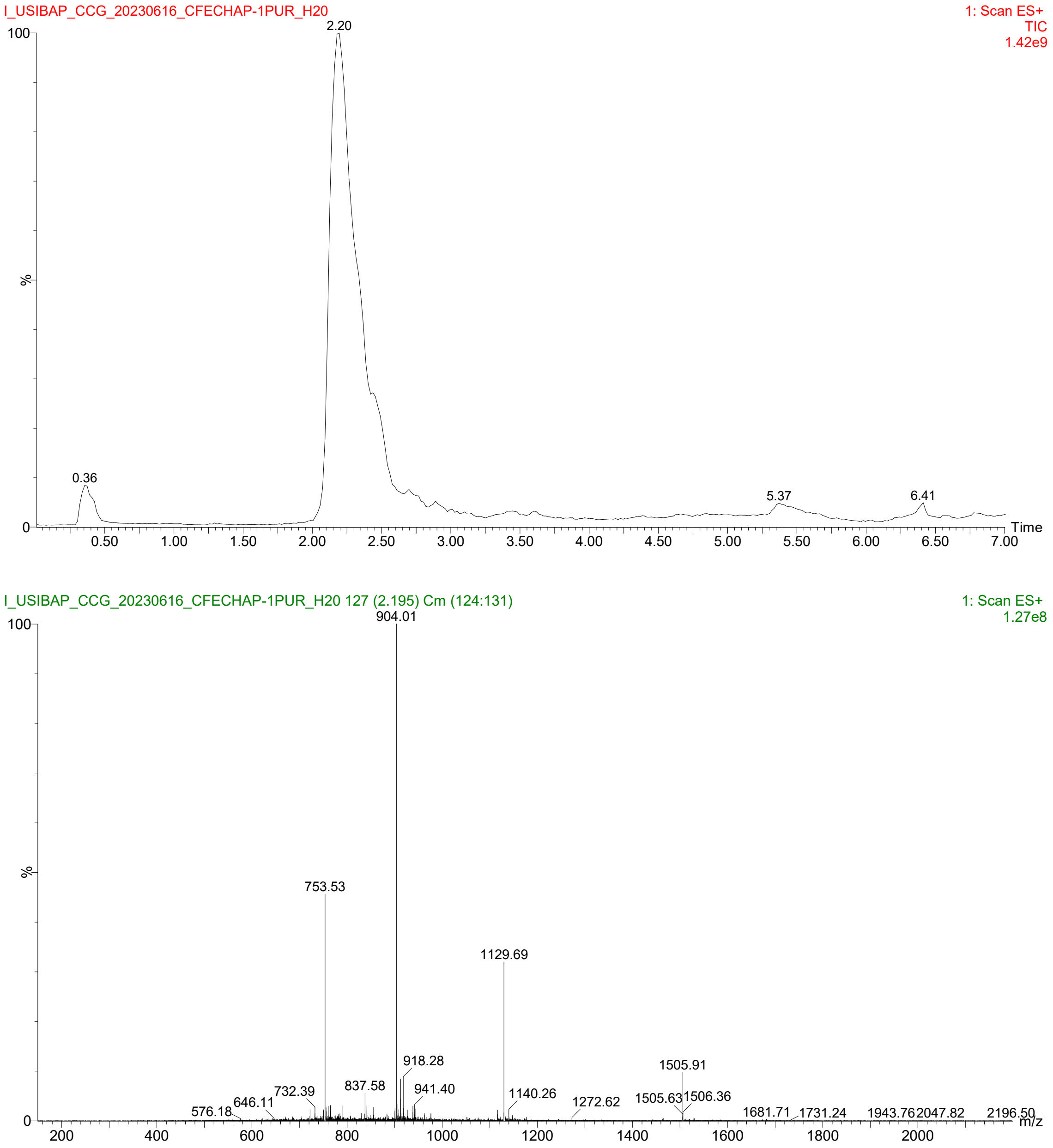

Figure S1. Primary structure, chemical formula, molecular weight, RP-HPLC chromatogram and ESI-MS spectrum of the **CFECHAP-1**. Calculated m/z: 1506.03 $[M+3H]^{3+}$, 1129.78 $[M+4H]^{4+}$, 904.02 $[M+5H]^{5+}$, 753.52 $[M+6H]^{6+}$; Found m/z: 1505.91 $[M+3H]^{3+}$, 1129.69 $[M+4H]^{4+}$, 904.01 $[M+5H]^{5+}$, 753.53 $[M+6H]^{6+}$

Biotinyl-PEG$_2$-HSTAcKRGHAhCitSRPVCitG-**Cys**AcKIHACitEIFDShCitGNPTVE**Cys** (Cyclic)

Chemical formula: $C_{191}H_{310}N_{62}O_{59}S_3$

MW: 4515.08 g/mol

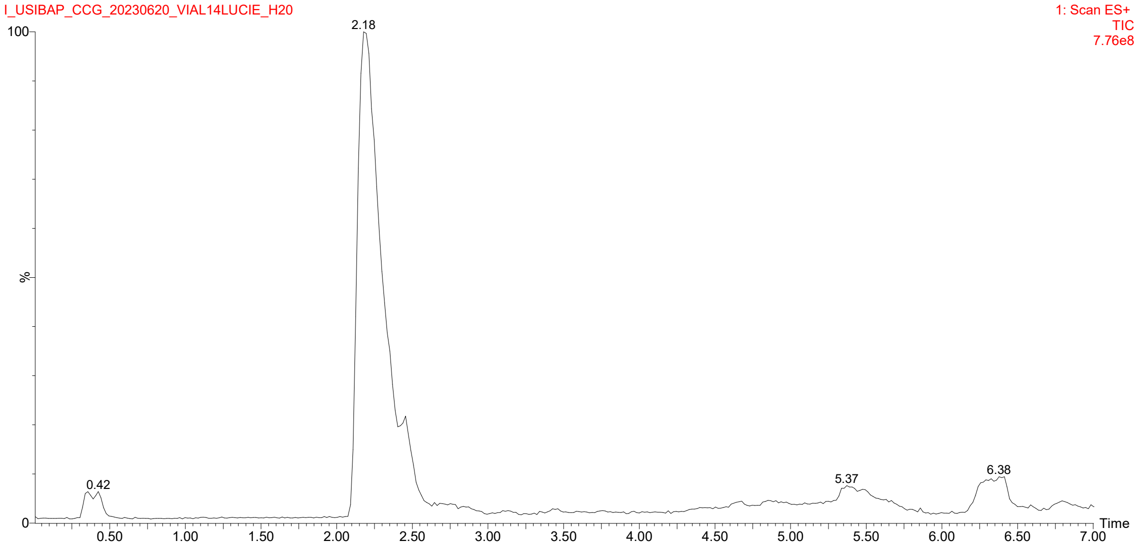

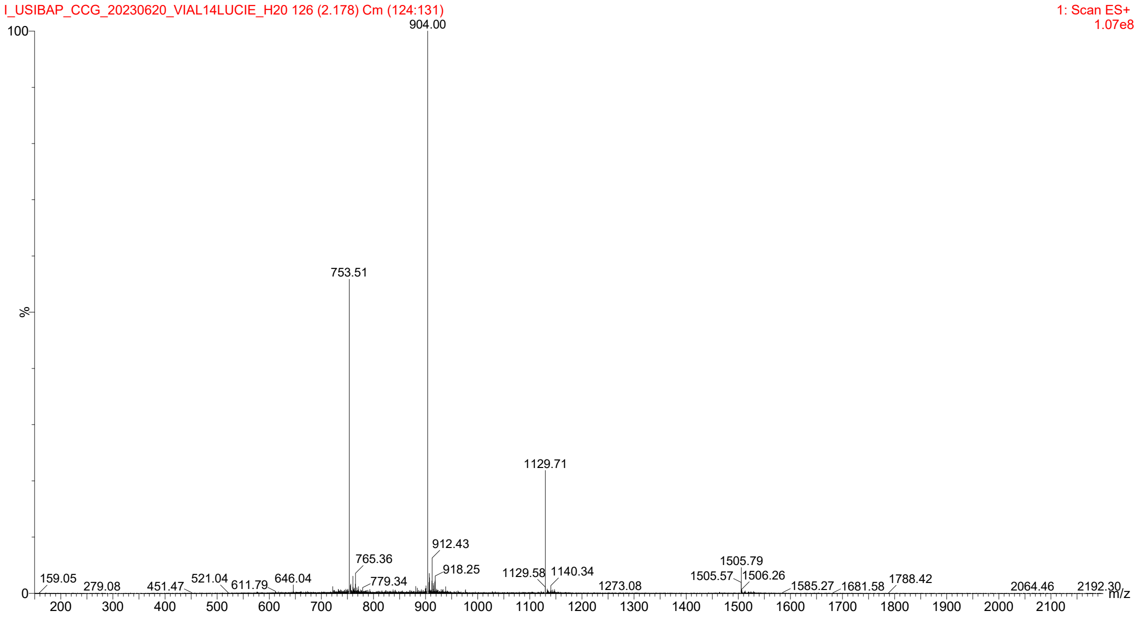

Figure S2. Primary structure, chemical formula, molecular weight, RP-HPLC chromatogram and ESI-MS spectrum of the **CFECHAP-2**. Calculated m/z: 1506.03 $[M+3H]^{3+}$, 1129.78 $[M+4H]^{4+}$, 904.02 $[M+5H]^{5+}$, 753.52 $[M+6H]^{6+}$; Found m/z: 1505.79 $[M+3H]^{3+}$, 1129.71 $[M+4H]^{4+}$, 904.00 $[M+5H]^{5+}$, 753.51 $[M+6H]^{6+}$

Biotinyl-PEG2-STSRSLYASSPGGVYATRSSAVRLCitS-**Cys**AcKIHACitEIFDShCitGNPTVE**Cys** (Cyclic)

Chemical formula: $C_{233}H_{377}N_{69}O_{77}S_3$
MW: 5473.10 g/mol

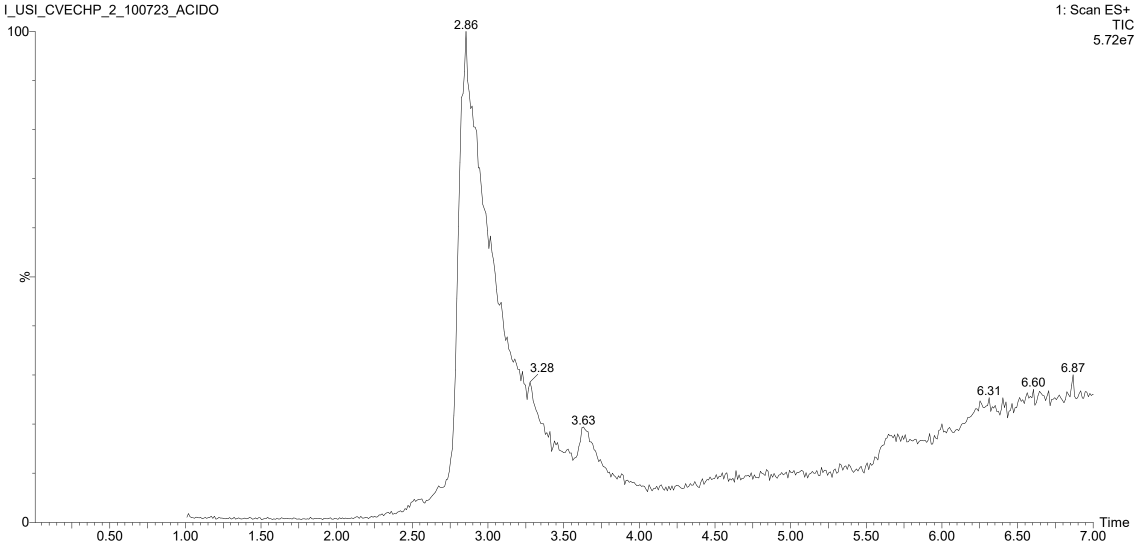

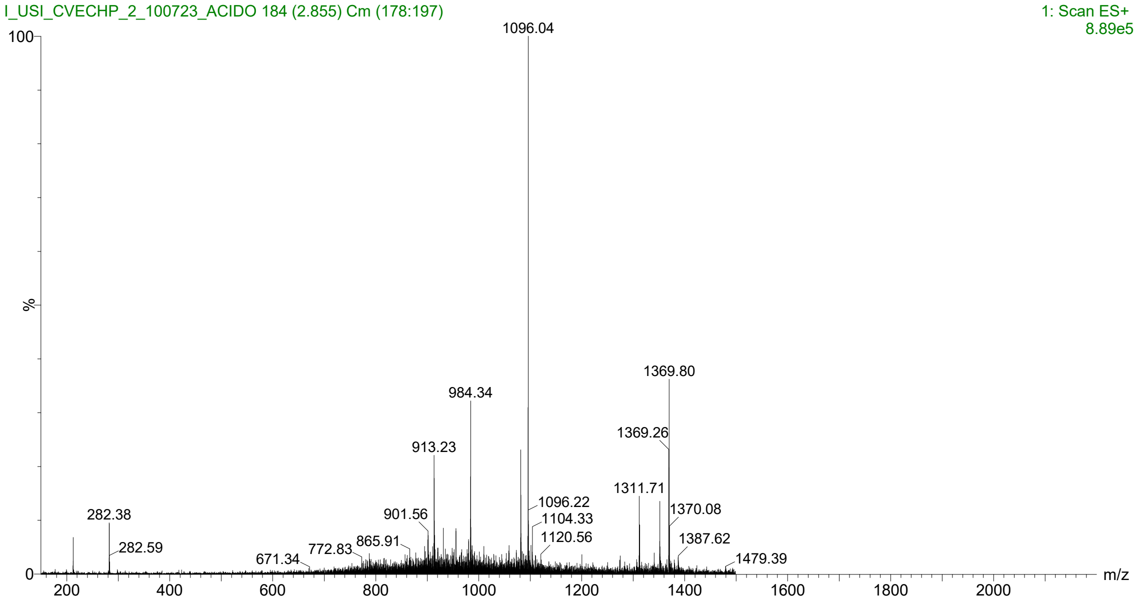

Figure S3. Primary structure, chemical formula, molecular weight, RP-HPLC chromatogram and ESI-MS spectrum of the **CVECHAP.** Calculated m/z: 1369.28 $[M+4H]^{4+}$, 1095.63 $[M+5H]^{5+}$, 913.19 $[M+6H]^{6+}$; Found m/z: 1369.80 $[M+4H]^{4+}$, 1096.04 $[M+5H]^{5+}$, 913.23 $[M+6H]^{6+}$

Biotinyl-PEG$_2$-KIHAREIFDSRGNPTVE-HQ**Cys**HQESTCitGRSRGR**Cys**GRSGS (Cyclic)

Chemical formula: $C_{200}H_{325}N_{71}O_{67}S_3$
MW: 4892,41 g/mol

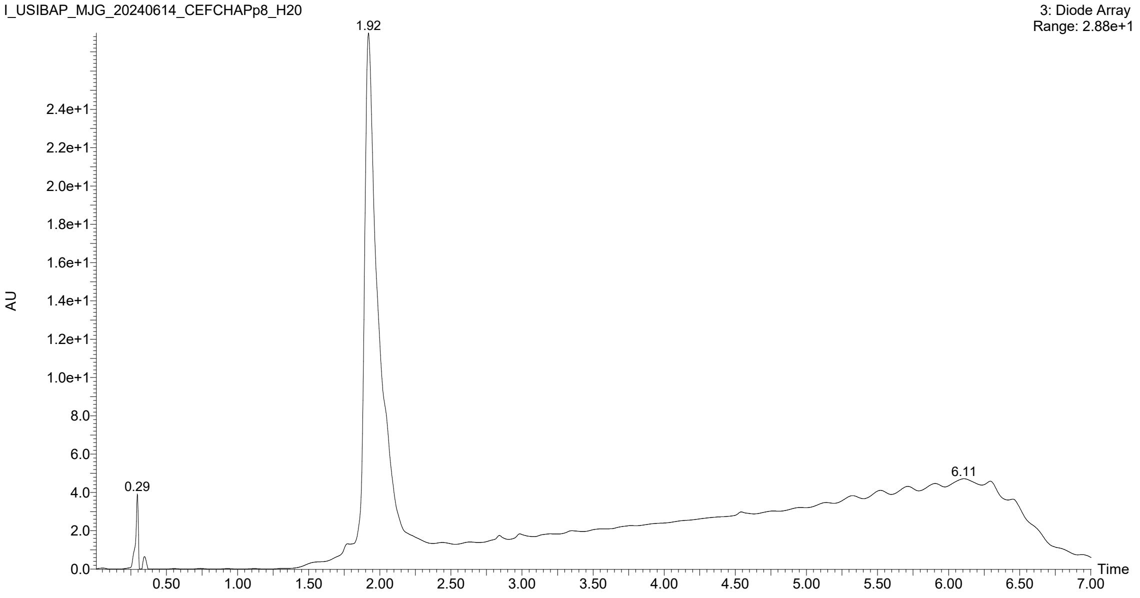

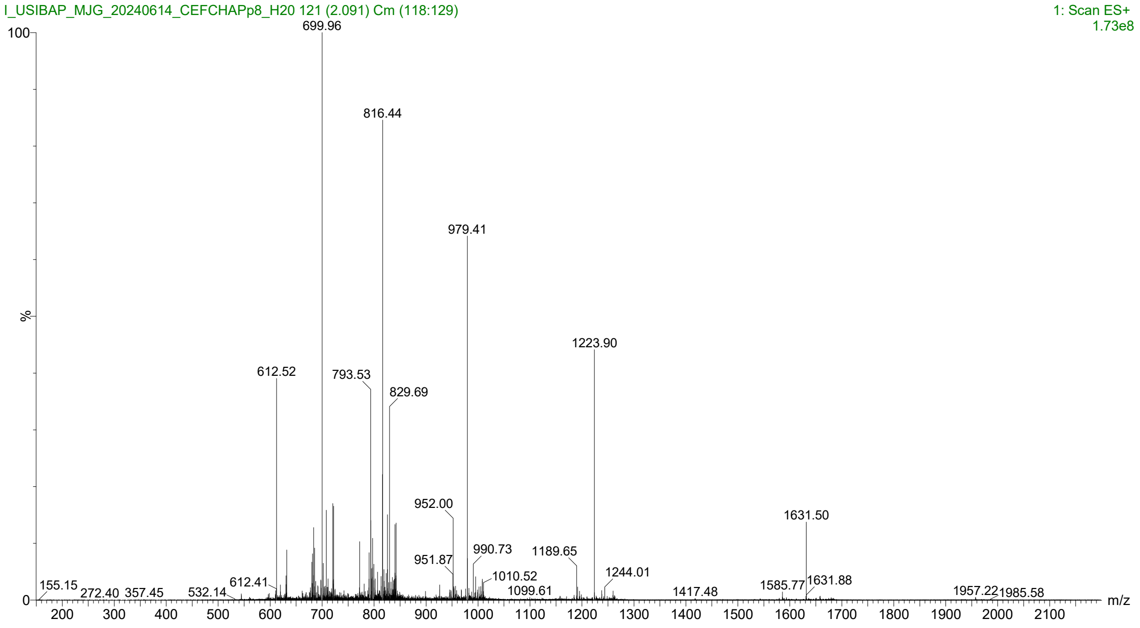

Figure S4. Primary structure, chemical formula, molecular weight, RP-HPLC chromatogram and ESI-MS spectrum of the **CEFCHAP.** Calculated m/z: 1631.79 $[M+3H]^{3+}$, 1224.10 $[M+4H]^{4+}$, 979.48 $[M+5H]^{5+}$, 816.40 $[M+6H]^{6+}$, 699.91 $[M+7H]^{7+}$, 612.55 $[M+8H]^{8+}$; Found m/z: 1505.79 $[M+3H]^{3+}$, 1129.71 $[M+4H]^{4+}$, 904.00 $[M+5H]^{5+}$, 753.51 $[M+6H]^{6+}$, 699.96 $[M+7H]^{7+}$, 612.52 $[M+8H]^{8+}$

Biotinyl-PEG2-HSTKRGHAKSRPVRG-**Cys**KIHAREIFDSRGNPTVE**Cys** (Cyclic)

Chemical Formula: $C_{185}H_{306}N_{64}O_{53}S_3$
MW: 4371.00 g/mol

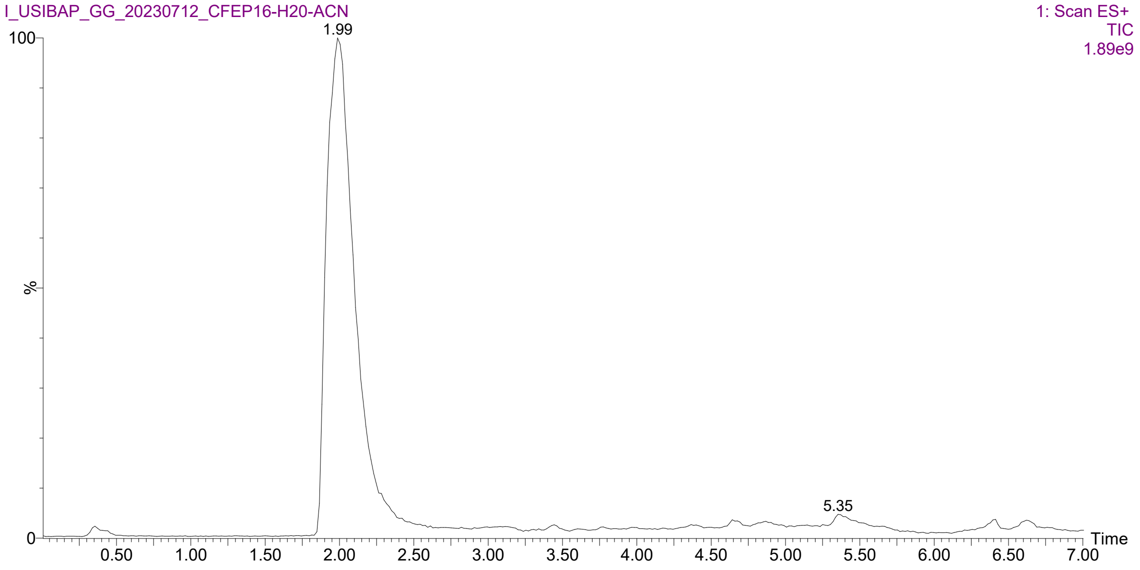

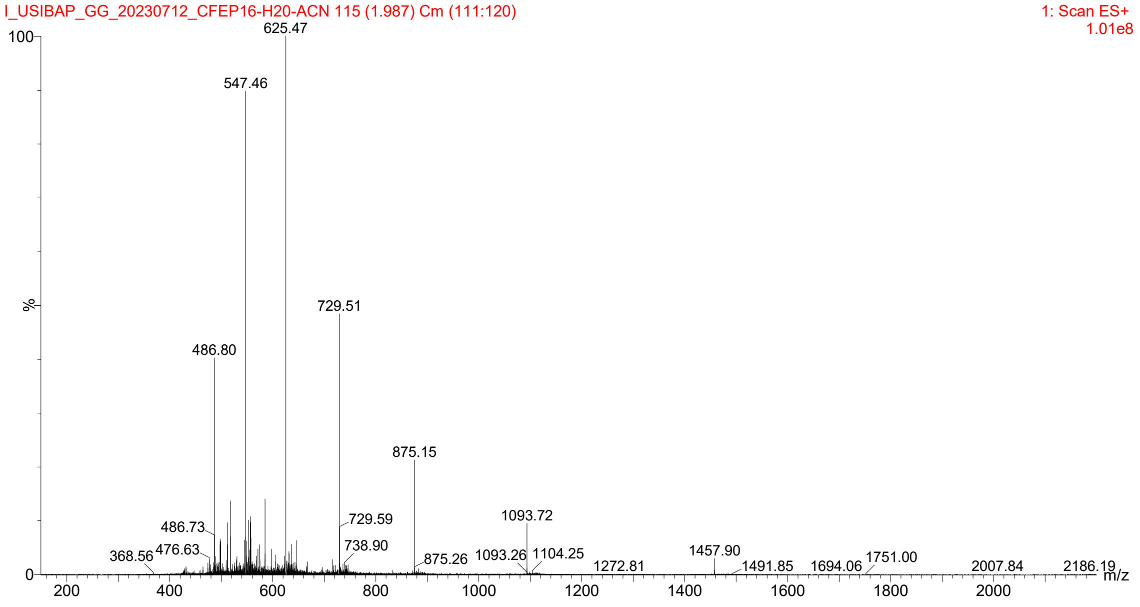

Figure S5. Primary structure, chemical formula, molecular weight, RP-HPLC chromatogram and ESI-MS spectrum of the **CFEP.** Calculated m/z: 1458.01 $[M+3H]^{3+}$, 1093.76 $[M+4H]^{4+}$, 875.21 $[M+5H]^{5+}$, 729.51 $[M+6H]^{6+}$, 625.44 $[M+7H]^{7+}$, 547.38 $[M+8H]^{8+}$, 486.67 $[M+9H]^{9+}$; Found m/z: 1457.90 $[M+3H]^{3+}$, 1093.72 $[M+4H]^{4+}$, 875.15 $[M+5H]^{5+}$, 729.51 $[M+6H]^{6+}$, 625.47 $[M+7H]^{7+}$, 547.46 $[M+8H]^{8+}$, 486.80 $[M+9H]^{9+}$

Biotinyl-PEG2-STSRSLYASSPGGVVYATRSSAVRLRS-**Cys**KIHAREIFDSRGNPTVE**Cys**(Cyclic)

Chemical formula: $C_{230}H_{376}N_{72}O_{73}S_3$
MW: 5414.08 g/mol

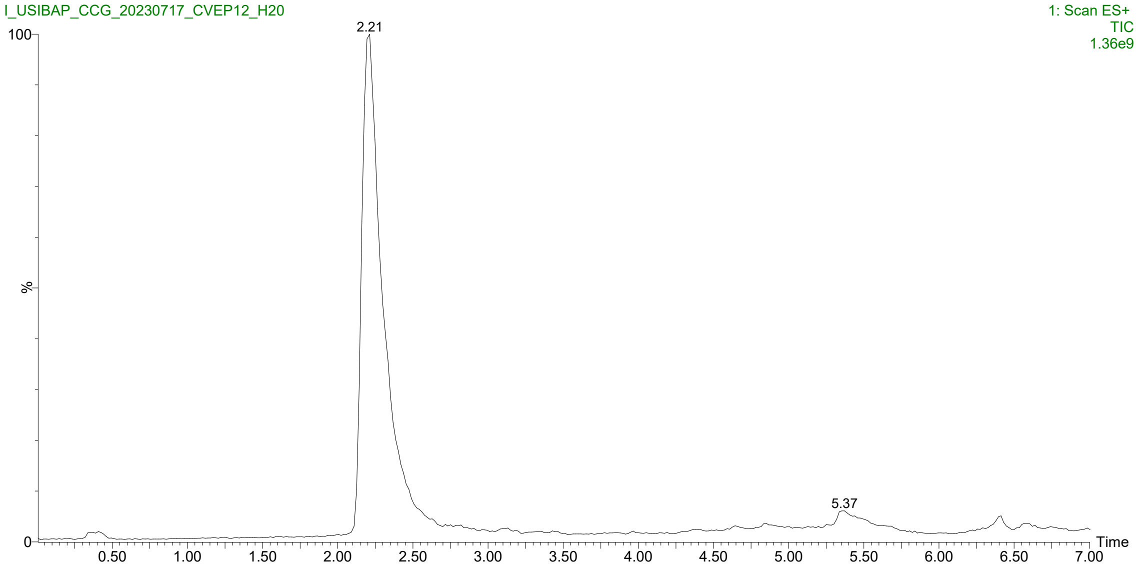

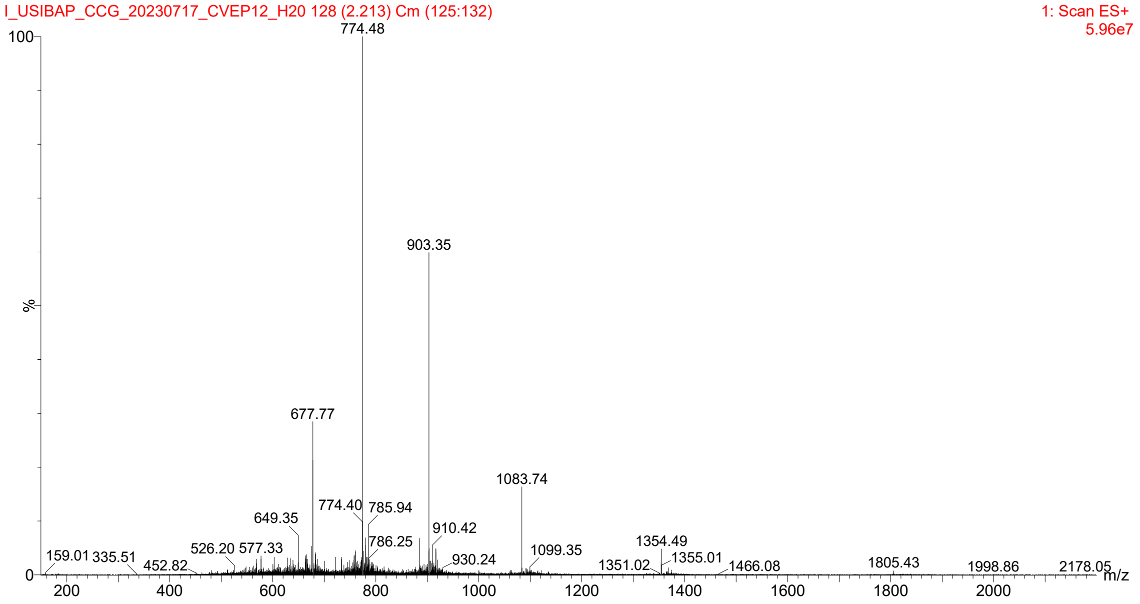

Figure S6. Primary structure, chemical formula, molecular weight, RP-HPLC chromatogram and ESI-MS spectrum of the **CVEP.** Calculated m/z: 1354.53 $[M+4H]^{4+}$, 1083.82 $[M+5H]^{5+}$, 903.35 $[M+6H]^{6+}$, 774.45 $[M+7H]^{7+}$, 677.77 $[M+8H]^{8+}$; Found m/z: 1354.49 $[M+4H]^{4+}$, 1083.74 $[M+5H]^{5+}$, 903.35 $[M+6H]^{6+}$, 774.48 $[M+7H]^{7+}$, 677.77 $[M+8H]^{8+}$

Biotinyl-PEG$_2$-KIHAREIFDSRGNPTVE-HQ**Cys**HQESTRGRSRGR**Cys**GRSGS (Cyclic)

Chemical formula: $C_{197}H_{324}N_{74}O_{63}S_3$
MW: 4833,39 g/mol

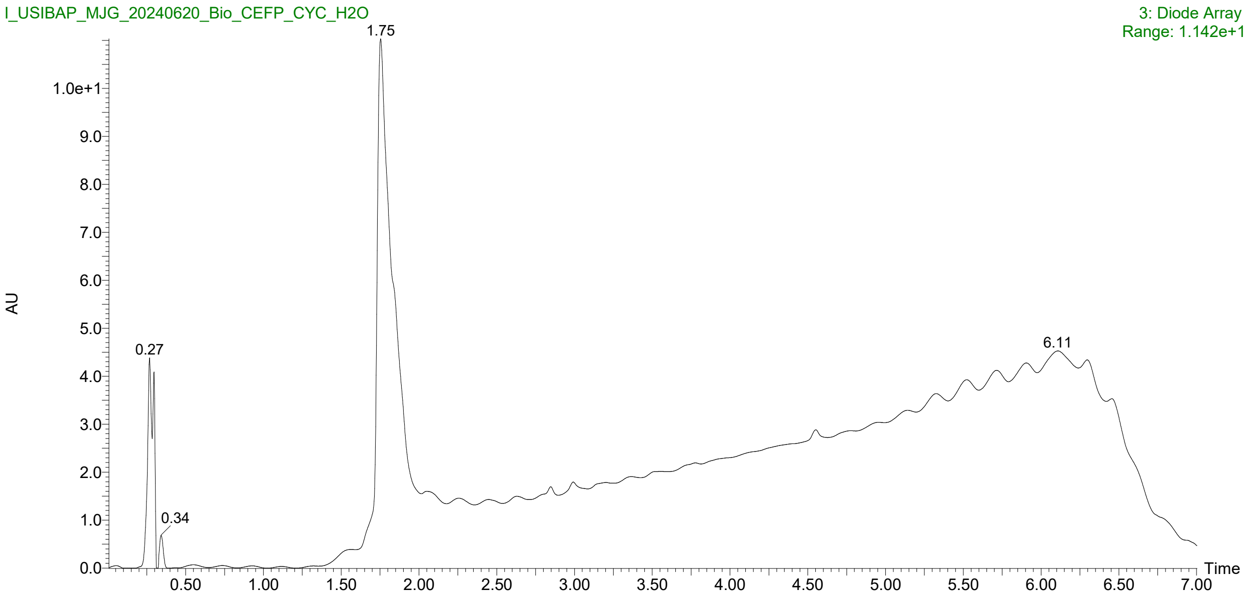

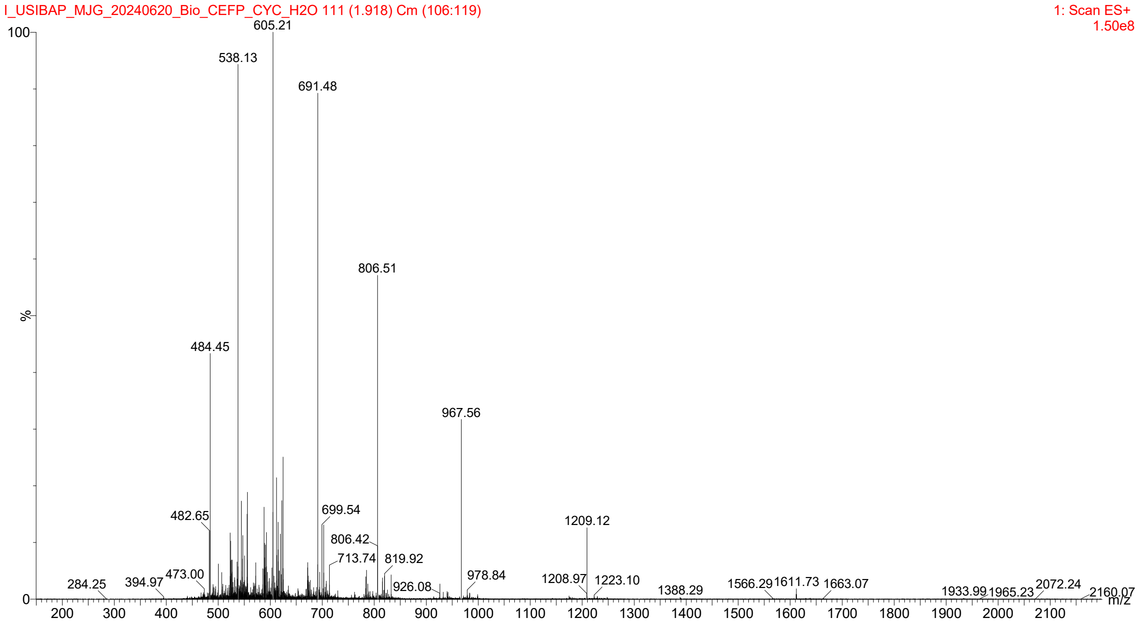

Figure S7. Primary structure, chemical formula, molecular weight, RP-HPLC chromatogram and ESI-MS spectrum of the **CEFP.** Calculated m/z: 1209.34 $[M+4H]^{4+}$, 967.67 $[M+5H]^{5+}$, 806.56 $[M+6H]^{6+}$, 691.48 $[M+7H]^{7+}$, 605.17 $[M+8H]^{8+}$, 538.04 $[M+9H]^{9+}$, 484.34 $[M+10H]^{10+}$; Found m/z: 1209.12 $[M+4H]^{4+}$, 967.56 $[M+5H]^{5+}$, 806.51 $[M+6H]^{6+}$, 691.48 $[M+7H]^{7+}$, 605.21 $[M+8H]^{8+}$, 538.13 $[M+9H]^{9+}$, 484.45 $[M+10H]^{10+}$
